# Morphine reduces local cytokine expression and neutrophil infiltration after incision

**DOI:** 10.1186/1744-8069-3-28

**Published:** 2007-10-02

**Authors:** J David Clark, Xiaoyou Shi, Xiangqi Li, Yanli Qiao, DeYong Liang, Martin S Angst, David C Yeomans

**Affiliations:** 1 Department of Anesthesia, Stanford University School of Medicine, Stanford, CA, USA; 2 Veterans Affairs Palo Alto Healthcare System, Palo Alto, CA, USA; 3 VAPAHCS Anesthesiology, 112A, 3801 Miranda Ave, Palo Alto, CA 94304, USA

## Abstract

**Background:**

Inflammation and nociceptive sensitization are hallmarks of tissue surrounding surgical incisions. Recent studies demonstrate that several cytokines may participate in the enhancement of nociception near these wounds. Since opioids like morphine interact with neutrophils and other immunocytes, it is possible that morphine exerts some of its antinociceptive action after surgical incision by altering the vigor of the inflammatory response. On the other hand, keratinocytes also express opioid receptors and have the capacity to produce cytokines after injury. Our studies were directed towards determining if opioids alter cytokine production near incisions and to identify cell populations responsible for producing these cytokines.

**Results:**

A murine incisional model was used to measure the effects of acute morphine administration (0.1–10 mg/kg) on nociceptive thresholds, neutrophil infiltration and cytokine production in hind paw skin 30 minutes and 2 hours after incision. Incised hind paws displayed profound allodynia which was reduced by morphine (0.1–10 mg/kg) in the 2 hours following incision. Skin samples harvested from these mice showed enhanced levels of 5 cytokines: IL-1β, IL-6, tumor necrosis factor alpha (TNFα), granulocyte colony stimulating factor (G-CSF) and keratinocyte-derived cytokine (KC). Morphine reduced these incision-stimulated levels. Separate analyses measuring myeloperoxidase (MPO) and using immunohistochemistry demonstrated that morphine dose-dependently reduced the infiltration of neutrophils into the peri-incisional tissue. The dose of morphine required for reduction of cytokine accumulation, however, was below that required for inhibition of peri-incisional neutrophil infiltration. Additional immunohistochemical studies revealed wound edge keratinocytes as being an important source of cytokines in the acute phase after incision.

**Conclusion:**

Acute morphine administration of doses as low as 0.1 mg/kg reduces peri-incisional cytokine expression. A reduction in neutrophil infiltration does not provide a complete explanation for this effect, and keratinocytes may be responsible for some incision area cytokine production. These studies suggest that morphine may alter the inflammatory milieu of incisional wounds, but these alterations do not likely contribute significantly to analgesia in the acute setting.

## Background

Surgically incised tissue provides an archetypical example of acute inflammation in which all classical signs and symptoms can be present: redness, swelling, heat, pain and reduced function. Investigators representing many disciplines have studied the mechanisms supporting inflammation in surgical wounds, and much has been learned about healing, infection and, more recently, pain related to wound inflammation. In fact, cytokines have long been recognized as controlling wound healing and resistance to wound infection [1,2]. It has been only recently, however, that we have recognized that cytokines produced in wounds or in peripheral neurons serving wounded tissues might influence pain and be legitimate targets for analgesic development. For example, the skin surrounding incisions and excisional biopsy sites has been shown to contain elevated amounts of several cytokines including interleukin-1β (IL-1β), interleukin-6 (IL-6), tumor necrosis factor-α (TNFα) and others [3,4]. Each of these cytokines has been observed to support enhanced nociceptive sensitivity in various rodent models [5-7]. Other cytokines such as IL-12 and IL-18 have also been shown to support nociception [8,9], though a few cytokines such as IL-4, IL-10 and IL-13 have anti-nociceptive effects [10]. The effects of cytokines may be model-specific, however. For example, a TNF receptor IgG fusion protein had no effect on incision-induced pain-related behaviors [11].

A recent report provided a somewhat more comprehensive profile of the types of cytokines expressed in response to skin incision, and the time course of those changes relative to nociceptive sensitization of the incised mouse hind paws [12]. This study identified enhanced levels of IL-1β, IL-6 and TNFα consistent with previous observations, but also suggested that granulocyte colony stimulating factor (G-CSF) and keratinocyte-derived cytokine (KC) were present in enhanced levels after incision. However, we have a poor overall understanding at this time of the relationship between this growing list of cytokines and incisional pain. Highlighting the difficulties in understanding the roles of cytokines in pain are observations that cytokines like IL-1β may have demonstrable roles in models of neuropathic and some types of inflammatory pain but not after incision [13]. A recent review discusses the complexity of cytokine biology as it relates to nociception [14].

Though largely unexplored, the presence of opioid receptors in skin and inflammation-related immunocytes suggests the possibility that acute or chronically administered opioids modulate incisional cytokine levels thereby influencing pain, swelling and other aspects of acute inflammation. Acutely administered opioids can, in fact, modify the degree of swelling caused by intradermal carrageenan injection [15,16]. Furthermore, opioid receptors are expressed by keratinocytes, the cells responsible for most skin cytokine production under resting conditions [17], and neutrophils which are acute phase immunocytes found in surgical wounds [18,19]. Reports over the past 20 years have described effects of endogenous and exogenous opioids in modulating both neutrophil migration and function [20-25] though these effects have not been studied in surgical wounds or with respect to pain. The principal goals of this study were, therefore, to determine if opioids like morphine could modulate wound cytokine levels when administered acutely, and whether any differences in cytokine levels identified might be attributable to opioid effects on wound area neutrophil infiltration versus enhanced production by resident cells.

## Results

### Acute morphine administration reduces mechanical allodynia after hind paw incision

**Figure 1:**
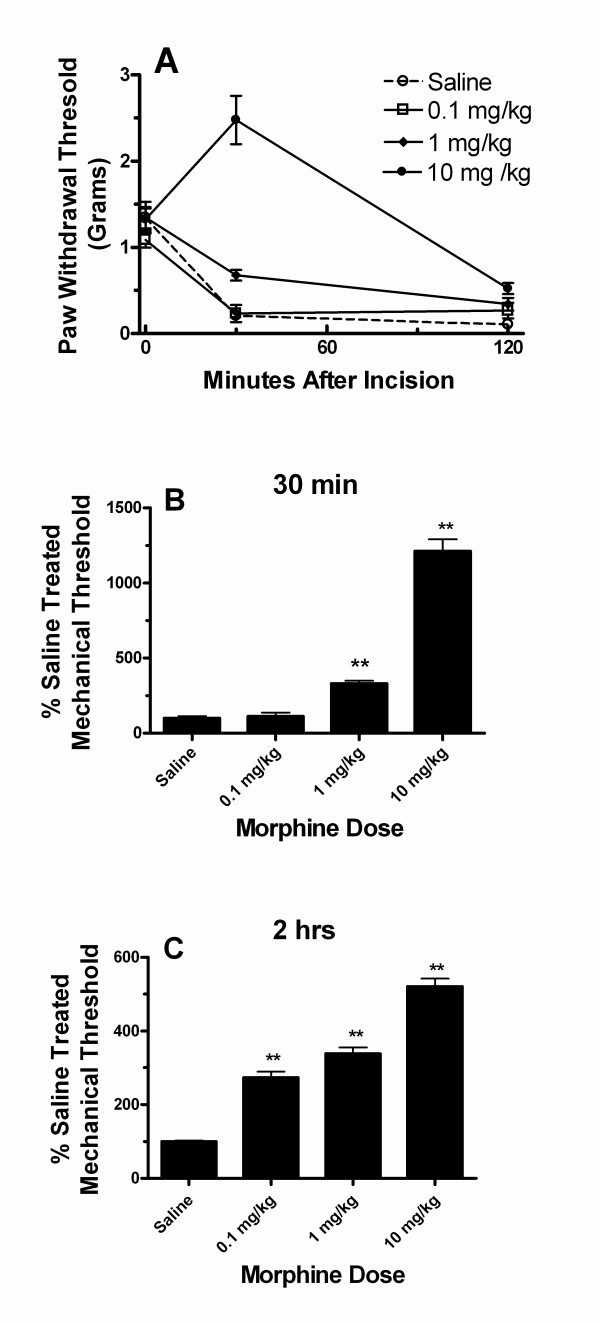
Morphine effects on post-incisional allodynia in mice. In these experiments the mechanical withdrawal thresholds of mice were measured before and at two time points after hind paw incision. Mice received saline or various doses of morphine immediately prior to making the incisions. Panel A displays the time course data. Panel B presents the mechanical thresholds for each group as a percent of the threshold for saline treated animals 30 minutes after incision. Panel C presents the mechanical thresholds for each group as a percent of the threshold for saline treated animals 2 hours after incision. Each group contained 5 mice. **p < 0.01 relative to saline treated animals.

Prior to investigating the effects of morphine on incisional cytokines, it was necessary that we establish dose-dependent effects of morphine on nociceptive sensitization resulting from incision. We therefore measured mechanical nociceptive thresholds before, 30 minutes and 2 hours after hind paw incision. Mice used in these experiments were given various doses of subcutaneous morphine taken from the C57BL/6 analgesia concentration range as established in other models [26,27]. We have previously established the duration of analgesia of the highest morphine dose given (10 mg/kg) to be approximately 2 hours, and that the analgesic effects are maximal within 30 minutes of administration [26]. The lower morphine doses used in these studies overlap the dose range employed clinically for the management of perioperative pain. As shown in Figure 1, morphine given prior to incision dose-dependently reduced mechanical allodynia for up to 2 hours after incision. The lowest dose of morphine used failed to reverse mechanical sensitization 30 minutes after hind paw incision. All doses of morphine provided a modest anti-allodynic effect 2 hours after incision.

### Acute morphine administration reduces peri-incisional cytokine production after hind paw incision

**Figure 2:**
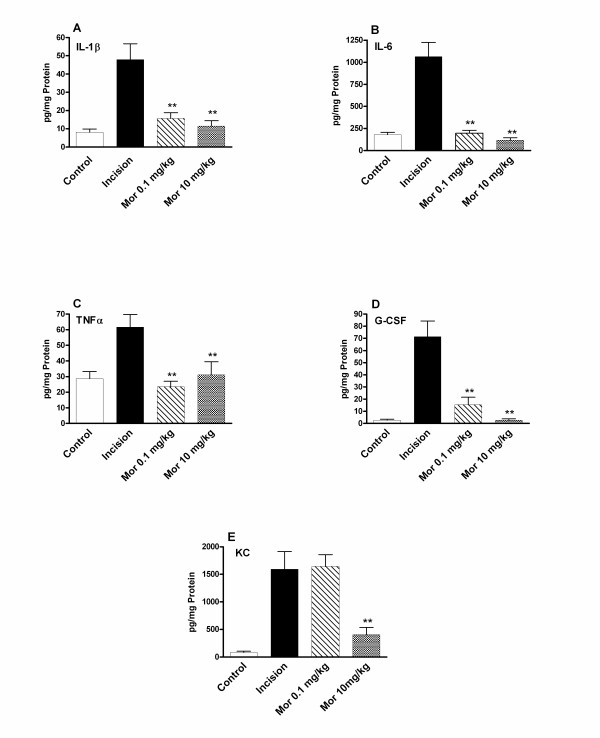
Hind paw skin cytokine levels at baseline and 2 hours after incision. For these experiments skin was harvested at baseline and 2 hours after incision near the end of the measured morphine analgesic effects. Mice were given saline or morphine of various doses immediately prior to the incisions being made. These analyses used tissue from 6 mice per treatment condition. The statistical significance of incised skin versus incised with prior morphine treatment was assessed. **p < 0.01.

In order to examine the possible immunomodulatory activity of morphine, we selected cytokines known to be present in increased amounts after hind paw incisions in mice based on our previous studies [12]. Thus the levels of IL-1β, IL-6, tumor necrosis factor alpha (TNFα), granulocyte colony stimulating factor (G-CSF) and keratinocyte-derived cytokine (KC) were followed. Measurements made at the 30 minute time point revealed that no elevations in wound area cytokine levels were yet measurable (data not shown). However, the data presented in Figure 2 show that the levels of the selected cytokines increased from 1.8 (TNFα) to >15-fold (G-CSF, KC) at 2 hours post incision. Other groups of animals were given morphine at either the highest dose used in the behavioral studies (10 mg/kg), or the lowest dose (0.1 mg/kg). Neither dose of morphine affected cytokine levels at 30 minutes after incision. On the other hand, at 2 hours morphine administered prior to incision strongly reduced cytokine levels at one or both of the doses tested. For the majority of cytokines the lower 0.1 mg/kg morphine dose had significant effects. The chemotactic cytokine KC, however, showed the same degree of induction after the injection of 0.1 mg/kg morphine as when saline was administered.

### Acute morphine administration reduces peri-incisional neutrophil infiltration after hind paw incision (myeloperoxidase assay, MPO)

**Figure 3:**
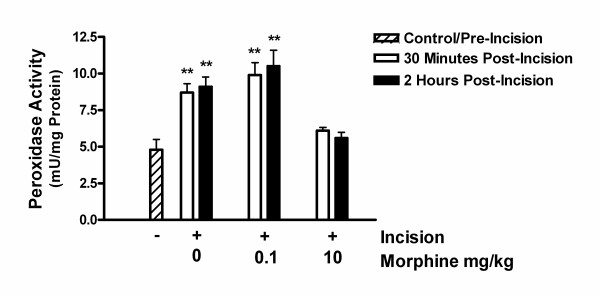
Myeloperoxidase levels in skin before and after hind paw incision. The skin of mouse hind paws was harvested before, 30 minutes after incision or 2 hours after incision. Incised animals were given saline or morphine of the indicated doses prior to incision. Eight control or incised samples per condition were used. Statistical analysis reflects differences in MPO levels versus control pre-incision levels. **p < 0.01.

Because of the large reductions in incised skin cytokine levels caused by acute morphine administration we proceeded to examine the infiltration of neutrophils, a potential source of wound cytokines. Wound skin edge neutrophil infiltration was assessed using a biochemical assay for myeloperoxidase (MPO) activity as has been employed for skin samples taken from excisional biopsy sites, scald wounds and after irritant injection [15,28,29]. In Figure 3 data are displayed demonstrating that even at 30 minutes after incision, MPO activity was measurably increased from baseline, and that 10 mg/kg but not 0.1 mg/kg morphine injection prior to incision strongly reduced the MPO activity at both time points.

### Acute morphine administration reduces peri-incisional neutrophil infiltration after hind paw incision (Immunohistochemistry)

**Figure 4:**
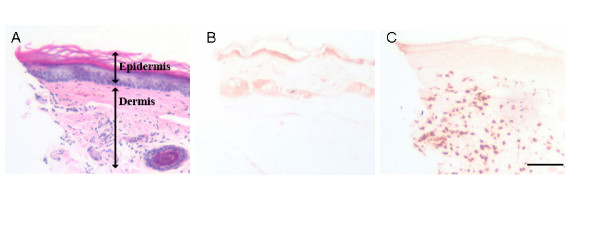
Peri-incisional infiltration of neutrophils, immunohistochemical appearance. Hind paw skin from incised mice was processed for the identification of neutrophils. Panel A displays an H&E stained section of plantar hind paw skin demonstrating a predominantly dermal cellular infiltrate 2 hours after incision. The dermal and epidermal layers are labeled. Panel B displays the appearance of non-incised hind paw skin stained with a neutrophil-specific antibody. Panel C displays a micrograph taken of incised skin stained for neutrophils 2 hours after incision. Note the abundance of darkly staining infiltrating neutrophils in the dermis of this section compared to that displayed in panel B. The scale bar in panel C is 150 μm in length. All micrographs were taken using 200× magnification.

**Figure 5:**
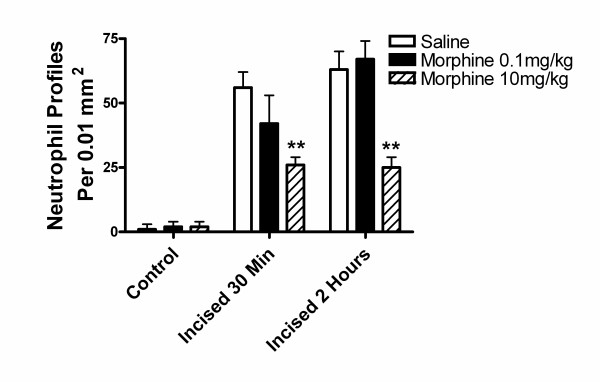
Peri-incisional infiltration of neutrophils, immunohistochemical quantification. The number of neutrophils in 0.01 mm^2 ^un-incised plantar skin or skin adjacent to incisions is presented. Animals were injected with saline or morphine 0.1 or 10 mg/kg prior to incision as indicated. For each condition 3–5 sections from 4 control or incised paws were analyzed. Statistical analysis was performed to detect differences between saline treated and morphine treated neutrophil counts. **p < 0.01.

In Figure 4 we present micrographs of wound edge tissue taken both before and 2 hours after incision. These were stained with either H&E or a neutrophil specific antibody. Examination of sections from incised versus control animals revealed the presence of a dense neutrophil infiltration after incision. Quantification of neutrophils in the wound edges revealed an approximate 40% reduction in neutrophil counts in the 10 mg/kg morphine treated animals at both 30 minutes and 2 hours when compared with samples from saline vehicle treated mice while (Figure 5). Consistent with the MPO data, 0.1 mg/kg had no significant effect.

### Incision leads to cytokine expression in the epidermis as well as in infiltrating neutrophils

**Figure 6:**
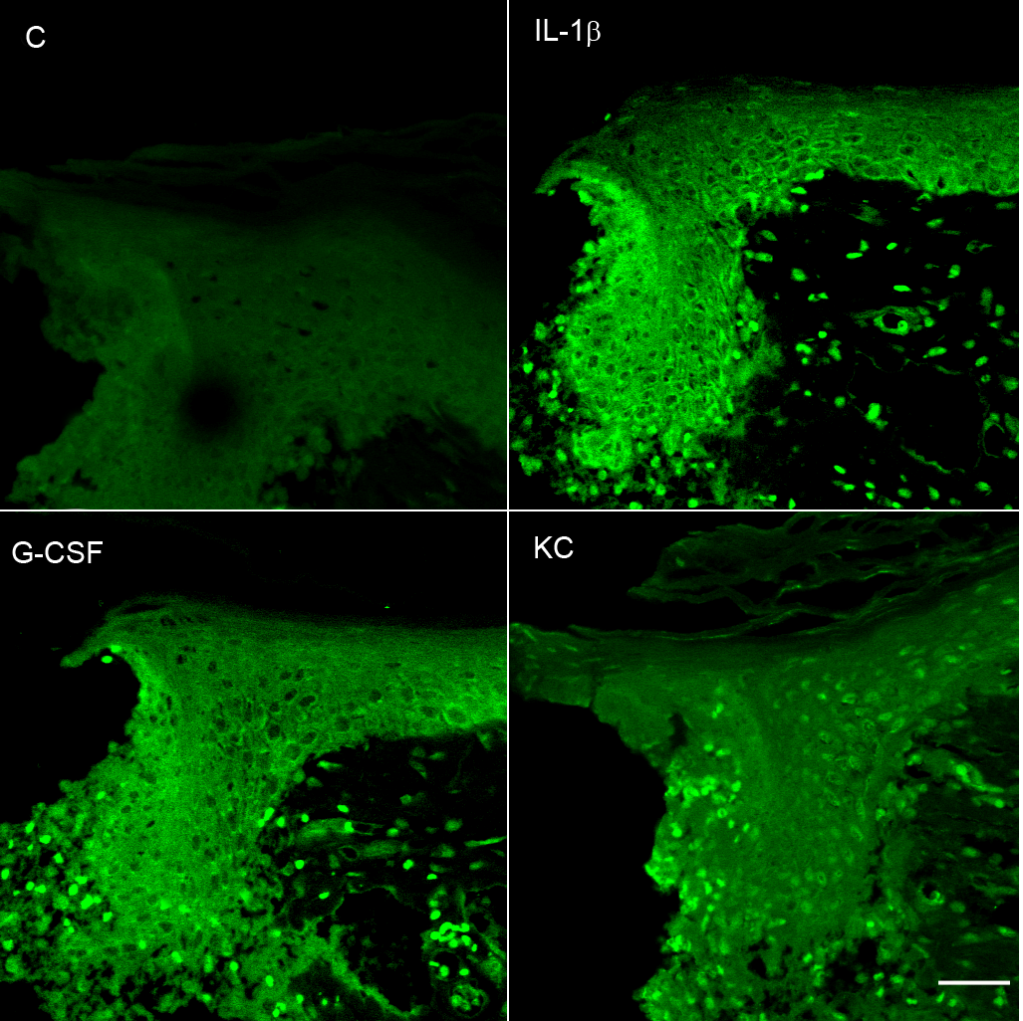
Confocal micrographs of incised skin. For these studies skin from the wound edges was collected 2 hours after the incisions were made. The tissue was sectioned and exposed to antibodies for IL-1β, G-CSF or KC then CY3 conjugated antibody allowing detection by fluorescence. Some sections were processed without primary antibody as controls (labeled "C"). These micrographs were taken under 400× magnification. The scale bar in the lower right hand (KC) panel is 100 μm in length.

**Figure 7:**
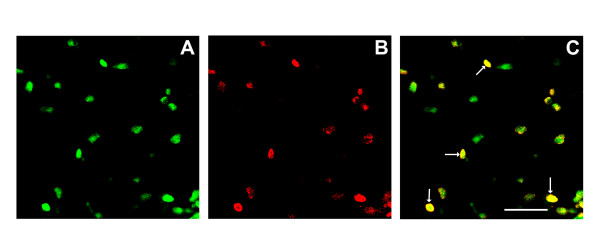
Confocal neutrophil-cytokine double labeling experiments using incised skin. For these studies skin from the wound edges was harvested 2 hours after incision. After sectioning, the tissue was exposed to anti-neutrophil and anti-IL-1β antibodies followed by the application of CY3 (green fluorescence, neutrophils, panel A) and FITC (red, IL-1β, panel B) conjugated secondary antibodies. Panel C presents the merged image with arrows pointing to several strongly double labeling cells. The scale bar in panel C is 50 μm in length. These micrographs were taken of dermal tissue under 630× magnification.

Because of the discordance in morphine effects on reducing wound edge cytokine levels versus effects on neutrophil infiltration, we attempted to identify additional cell types near the incisions producing cytokines. For these studies we chose to stain skin for an interleukin showing a large changes in expression after incision (IL-1β), G-CSF as it is proposed to have a role in supporting anti-inflammatory processes in the innate and adaptive immune systems [30], and KC since its sensitivity to morphine was distinct from the other 4 cytokines studied. In each case we identified strong expression of these cytokines in the keratinocytes comprising the epithelium of the skin adjacent to the 2 hour old incisional wounds (Figure 6). Also evident in Figure 6 are infiltrating immunocytes in dermal tissue expressing each of these cytokines. Double staining experiments established these to be neutrophils. Figure 7 demonstrates dermal peri-incisional neutrophils expressing IL-1β. Images from other double labeling experiments involving anti-neutrophil along with anti-G-CSF and anti-KC antibodies were similar in that neutrophils were observed to express each of these cytokines as well (data not shown).

## Discussion

In these investigations we set out to determine whether morphine administered acutely would alter skin incision cytokine levels, neutrophil infiltration or the nociceptive sensitization which accompanies this type of tissue trauma. The generation of cytokines after incision has importance in several areas including the maintenance of pain, combating infection and controlling wound healing. Factors significantly affecting cytokine production may, therefore, impact several processes important in the perioperative period. Consistent with our hypothesis, the acute pre-incisional administration of morphine reduced wound area cytokine production. Given the large effects on cytokine production, we went on to determine if morphine was reducing neutrophil migration to the wound area. Both a biochemical assay involving the quantification of myeloperoxidase (MPO) and immunohistochemical examination showed morphine mediated reductions in wound area neutrophil presence 30 minutes and 2 hours after hind paw incisions when a morphine dose of 10 mg/kg was administered. However, with the exception of KC levels, peri-incisional cytokine levels were reduced by the 100-fold lower 0.1 mg/kg morphine dose, a dose at which neutrophil infiltration was unaffected. Therefore, the morphine-induced reductions in cytokine levels cannot be due to reduced neutrophil infiltration alone. In fact, there appears to be a significant temporal delay between the arrival of neurtrophils in the wound and the production of cytokines (Figures 2 and 3). Keratinocytes were identified as an alternative source of peri-incisional cytokines.

Ours are not the first data supporting the notion that an acutely administered exogenous opioid can reduce inflammation and the abundance of key inflammatory mediators. Fecho et al. reported recently that the hind paw swelling induced by carrageenan injection could be reduced by morphine, and that MPO levels could be lowered by this opioid as well [15]. Systemic TNFα and IL-6 levels were not found to be changed in these experiments though local skin levels were not assessed. Similar studies were undertaken by Pourpak et al. who reported that high (7 mg/kg) but not low (1 mg/kg) doses of systemic morphine reduced hind paw swelling after carrageenan injection. In this study, however, the high dose of morphine in combination with carrageenan actually increased serum IL-1β levels [16]. Our results are somewhat different in that morphine consistently reduced local tissue interleukin levels and nociceptive sensitization across a wide dose range. One explanation for the differences in the reported results could be that we measured cytokine levels at the actual site of tissue trauma. These levels may be driven by factors partially separate from those controlling systemic levels.

Aside from the pain related studies, the influences opioids have on neutrophil function have long been of interest particularly as the findings might help us to understand possible differences in the risk of infection amongst drug abusers, burn victims and postoperative patients. One of the earliest studies conducted in the perioperative setting showed that relatively large (for humans) doses of morphine (0.5–1.1 mg/kg) inhibited neutrophil chemotaxis [24]. Our MPO data from mice having received the larger 10 mg/kg morphine dose prior to incision are consistent with these observations. Later studies confirmed these findings and showed definitively that both μ- and δ-opioid receptors likely mediate these opioid effects [20,22]. A larger field of work suggests that morphine and other opioids alter various aspects of immune system functioning including infection fighting, tumor cell destruction, lymphocyte proliferation, and apoptosis [31-33]. Importantly, morphine has been demonstrated to reduce neutrophil phagocytic activity [20,25,34-36]. Thus morphine may affect neutrophil function in many ways other than wound area infiltration as followed in our experiments. For example, morphine may reduce cytokine production in neutrophils infiltrating peri-incisional skin though we have not yet tested this hypothesis.

While the focus of most work concerning opioid effects in wounds has centered on neutrophils and other immunocytes, resident cells such as keratinocytes also express functional μ-opioid receptors [17,37]. The keratinocytes of the epidermis also produce endogenous opioid peptides, though little study has been devoted to the ability of these peptides to alter skin cytokine levels [17,38]. Because low dose (0.1 mg/kg) morphine strongly reduced incision-induced cytokine level increases for most of the measured cytokines but failed to reduce neutrophil infiltration at this dose, we considered other cell types as sources of wound cytokines. The results shown in Figure 6 demonstrate that cytokine production is strongly up-regulated within 2 hours of incision in the epidermis. Within the epidermis, it is primarily the basal and suprabasal layer keratinocytes which express μ-opioid receptors [37,39,40]. These receptors have been linked with control of cell migration in cultures of keratinocytes [41]. Furthermore, keratinocytes have been demonstrated to be important sources of skin cytokines in other models. For example, keratinocytes appeared to be an important source of IL-10 in a separate murine incisional wound model [42]. Also, keratinocytes stimulated with the nociception related peptides substance P (SP) and calcitonin gene related peptide (CGRP) express enhanced levels of IL-1α, IL-8 and TNFα [43]. It is possible, therefore, that morphine controls skin cytokine levels by virtue of a direct interaction with keratinocytes or by reducing the release of substance P and CGRP from cutaneous nociceptors, which, in turn, reduces the production of cytokines by resident skin cells. Further experiments performed in well-defined cell culture systems may tell us whether endogenous or exogenous opioids alter keratinocyte cytokine production.

Interestingly, the one cytokine measured not displaying decreased abundance in the incised skin of mice after low dose (0.1 mg/kg) morphine administration was KC. This low morphine dose also did not reduce neutrophil infiltration. This cytokine has powerful neutrophil chemoattractant properties in mouse skin, and is thought to be responsible for much of the early neutrophil infiltration in surgical and scalded skin injury models [44,45]. Though we did not specifically investigate the hypothesis, it appears possible that production of this cytokine is under separate control from the other incision related cytokines and is one of the factors responsible for the recruitment of neutrophils into hind paw incisional wounds. With respect to nociception, however, other investigators have demonstrated that KC induced neutrophil infiltration by itself is not sufficient to cause thermal or mechanical hyperalgesia in rodent hind paw tissue [46].

Our data also allow us to comment on the likely importance of the group of cytokines we studied to allodynia after incision. First, at 30 minutes after incision allodynia was maximal yet cytokine levels had not yet changed. Furthermore, Figure 1 demonstrates that at the 2 hour time point mechanical thresholds are reversed only to about 40% of baseline with 10 mg/kg morphine and are reversed to an even lesser extent by 0.1 mg/kg morphine despite skin cytokine levels being at essentially baseline levels (Figures 1, 2). Thus it seems very likely that factors other than cytokines are strong contributors to allodynia after incision at these acute time points. Some locally produced factors which are more likely to contribute to acute incisional wound nociceptive sensitization include hydrogen ion, ATP, neuropeptides, prostaglandins, bradykinin, serotonin and other molecules. By extension, it appears unlikely that analgesic therapies directed only at reducing wound levels of the cytokines we studied would have a high degree of efficacy within the first few hours after incision. As mentioned in the introduction, the roles of TNFα and IL-1β in supporting mechanical sensitization after incision have been questioned [11,13]. It needs to be recognized, however, that we measured only mechanical thresholds, and that the cytokines studied might be involved to a greater extent in controlling thermal or other types of nociceptive sensitization. This seems to be the case for NGF which supports thermal to a greater extent than mechanical sensitization in tissue surrounding incisions [11].

Rather than considering incisional wounds as static lesions, we may need to consider the possibility that the wound area mechanisms supporting nociception change rapidly over the fist few hours to days and that the pharmacology of nociception may change over this time course as well. In this regard it is notable that a complement fragment C5a antagonist was more effective at reversing wound area mechanical allodynia 24 hours after incision when the levels of several C5a-regulated cytokines were at maximum than 2 hours after incision [12]. In the present study the 0.1 mg/kg dose of morphine had no effect at 30 minutes but did modestly reverse allodynia at the 2 hour time point where the same dose strongly reduced wound area cytokines. Cytokine participation in supporting nociception may be very different at 24 hours following incision. Our data are also consistent with the hypothesis that it takes hours for cytokines to accumulate in wounds to the point where they are important contributors to nociceptive sensitization. Additional experiments employing selective opioid receptor antagonists, cytokine neutralizing antibodies and mediator knockout mice at a wider range of time points will be necessary to fully define the role of cytokines in post-incisional nociceptive sensitization.

## Conclusion

We currently focus on the direct neuromodulatory effects of opioids to explain the analgesic effects of these drugs after surgery. Indeed there are substantial data supporting both central and peripheral neuronally mediated opioid analgesic effects. It is also true that a growing body of literature supports pro-nociceptive functions for a range of cytokines. In these studies we were able to show that while morphine can reduce these wound area cytokines, such reductions contribute only minimally to anti-nociception in the acute setting. Aside from potentially affecting nociception, the inflammation altering effects of exogenous opioids may affect resistance to infection and perhaps the healing characteristics of these wounds. Future studies might be directed at addressing all three of these possibilities across a broader time course after surgical incision.

## Methods

### Animal use

All experimental protocols were reviewed and approved by Veterans Affairs Palo Alto Healthcare System institutional animal care and use committee prior to the initiation of work. Male mice 12–14 weeks old of the C57Bl/6J strain were kept under standard conditions with a 12 h light/dark cycle and allowed food and water ad libitum. Mice were obtained from Jackson Laboratories (Bar Harbor, MA) and were kept in our animal facility a minimum of 1 week prior to use in experiments.

### Hind paw incision

The hind paw incision model was used as modified for mice [47]. We have used this model previously in order to study cytokine levels following incision [12]. Briefly, mice were anesthetized using isoflurane. After sterile preparation, a 0.5 cm incision was made with a #15 scalpel blade on the plantar surface of one hind paw. This incision was sufficiently deep to divide the plantaris muscle longitudinally. After briefly holding pressure to stop any active bleeding, a single 6-0 nylon suture was placed and antibiotic ointment applied. Mice were then returned to their cages on soft bedding. Nociceptive testing and tissue harvest took place 30 minutes or 2 hours after incision.

### Drug administration

Morphine or saline vehicle was administered immediately prior to incision. To accomplish this, 100 μl 0.9% NaCl with or without morphine (0.1–10 mg/kg, Sigma Chemical, St. Louis, MO) was injected subcutaneously into the skin of the back.

### Nociceptive testing

Mechanical allodynia was assayed using nylon von Frey filaments according to the "up-down" algorithm described by Chaplan et al. [48] as we have used previously to detect allodynia in mice [12,49,50]. In these experiments mice were placed on wire mesh platforms in clear cylindrical plastic enclosures 10 cm in diameter. After 15 minutes of acclimation, fibers of sequentially increasing stiffness were applied 1 mm lateral to the wound edge, pressed upward to cause a slight bend in the fiber and left in place 5 sec. Withdrawal of the hind paw from the fiber was scored as a response. When no response was obtained the next stiffest fiber in the series was applied to the same paw; if a response was obtained a less stiff fiber was applied. Testing proceeded in this manner until 4 fibers had been applied after the first one causing a withdrawal response allowing the estimation of the mechanical withdrawal threshold [51].

### Cytokine analysis

To obtain skin samples for cytokine quantification animals were first sacrificed by CO2 asphyxiation and an ovular patch of full-thickness skin providing 1.5 mm margins surrounding the hind paw incisions was collected by dissection. Each such patch contained approximately 12 mg tissue. These samples were placed immediately into ice cold 0.9% NaCl containing a cocktail of protease inhibitors (Complete™, Roche Applied Science, Indianapolis, IN). Approximately 750 μl inhibitor containing saline was used per 25 mg tissue. The samples were homogenized using a Polytron device (Brinkman Instruments Inc., Westbury, NY), then centrifuged for 10 min at 12,000 times gravity at 4°C. Supernatant fractions were kept frozen at -80°C until use. An aliquot was subjected to protein assay (DC Protein Assay, Bio-Rad Laboratories, Hercules, CA).

For the cytokine assays, custom Bio-Rad (Bio-Rad laboratories, Hercules, CA) Bio-Plex cytokine analysis kits were used in conjunction with the Bio-Plex system array reader according to the manufacturer's directions as described previously [12]. The specific cytokines were chosen based on our previously reported results. Samples were diluted 1:2 prior to analysis, and all samples were run in duplicate in each assay. We demonstrated previously that the dynamic range of sensitivity of this assay was sufficient to accurately measure both basal and incision-stimulated levels of the chosen cytokines [12]. Standard curves for each of the analyzed substances were included in each run, and sample concentrations were calculated using Bio-Plex Manager software.

### Histological analysis

We have previously reported our methods for the immunohistochemical analysis of incised mouse paw skin [12]. For these analyses mice were sacrificed using carbon dioxide asphyxiation which was followed by intracardiac perfusion of 20cc 0.9% NaCl followed by 20 ml of 10% neutrally buffered formalin. Hind paws were then removed and incubated in 10% buffered formalin for 24 hours. After overnight decalcification, tissue was processed for paraffin sectioning in automated fashion (Tissue Tek VIP, Miles Scientific, Naperville, IL). Following embedding, 6.5 μM slices were made, placed on slides and incubated for 20 min at 55°C to improve adherence. Some sections were stained with hematoxylin and eosin (H&E). These were viewed and imaged using an Olympus BH-2 microscope and Spot RT digital imaging equipment (Diagnostic Instruments, Sterling Heights, MI).

Other paraffin sections were processed for the identification of neutrophils. Paraffin was removed with graded xylenes then re-hydrated in ethanol. Endogenous peroxidase was quenched by incubation in 0.3% H_2_O_2 _for 30 minutes. Blocking of these sections took place for 3 hours in 5% normal rabbit serum followed by exposure to rat anti-mouse neutrophil antibody 1:5000 (MCA7771GA, Serotec, Raleigh, NC). After additional rinsing, ABC reagents were applied according to the manufacturers directions (Vector labs, Burlingame, CA) followed by the application of coverslips using Permount (Biomedia, Foster City, CA). Control experiments included incubation of slices in primary or secondary antibody-free solutions both of which resulted in low intensity non-specific staining patterns in preliminary experiments. The specificity of the anti-neutrophil antibody was confirmed by Western blotting.

The quantification of neutrophils was done with the counter blind to the treatment condition. After collecting images using 100–400× total magnification, the number of neutrophils in a rectangle with boundaries from the skin surface to a point 200 μm deep and from the lateral wound edge inward 50 μm were counted.

Additional experiments involved confocal imaging. For these studies blocking of paraffin sections took place overnight at 4°C in tris buffered saline (TBS) containing 5% dry milk, followed by exposure to one of the following primary antibodies overnight at 4°C in milk-TBS: polyclonal anti-IL1β, 1:500 (Santa Cruz Biotechnology, Santa Cruz, CA); polyclonal anti-G-CSF, 1:200 (Abcam Inc.); polyclonal anti-KC/GROα, 1:50 (Santa Cruz). Sections were also exposed to anti-mouse neutrophil antibody 1:5000 (Serotec). Sections were then be rinsed and transferred to milk-TBS containing fluorescein conjugated secondary antibodies 1:300–1:500 (Jackson ImmunoResearch Laboratories, West Grove, PA) and incubated for another 1 hour. After washing, coverslips were applied. Confocal laser-scanning microscopy was carried out using a Zeiss LSM/510 META microscope. Images were stored on digital media. Control experiments included incubation of slices in primary and secondary antibody-free solutions both of which lead to low intensity non-specific staining patterns in preliminary experiments.

### Myeloperoxidase (MPO) assay

MPO activity was measured as a biochemical index of neutrophil recruitment in the wound edge samples [52]. Excised skin samples were washed in PBS and homogenized in 1 ml 50 mM potassium phosphate-buffer solution with 0.5% hexadecyl trimethyl ammonium bromide (Sigma Chemical Co., St. Louis, MO) and 5 mM EDTA. The samples were then homogenized as above and centrifuged at 12,000 rpm at 4°C. MPO activities in the supernatants were then assayed using a peroxidase assay kit (Anaspec, San Jose, CA) along with supplied standards according to the manufacturer's instructions. The data were expressed as MPO activity in mU per mg protein in the supernatant samples.

### Statistical analysis

Analysis of repeated parametric measures was accomplished using a one-way ANOVA analysis of variance followed by post-hoc Dunnett's testing or a two-way ANOVA followed by Bonferroni testing. For simple comparisons of 2 means t-testing was employed. A value of p < 0.05 was taken to be significant. All data are presented as means +/- S.E.M. unless otherwise noted.

## List of Abbreviations

ANOVA Analysis of variance

S.E.M. Standard error of the mean

MPO Myeloperoxidase

EDTA Ethylenediamine tetra-acetic acid

TBS Tris-buffered saline

NGF Nerve growth factor

TNF Tumor necrosis factor

ATP Adenosine triphosphate

KC Keratinocyte derived cytokine

CGRP Calcitonin gene-related peptide

GCSF Granulocyte colony stimulating factor

## Competing interests

The author(s) declare that they have no competing interests.

## Authors' contributions

JDC: This is the senior architect of the project who designed most experiments, wrote most of the manuscript and obtained funding for the project.

DL: This person performed all behavior testing.

YQ: This person performed all cytokine assays

XL: This person did all immunohistochemistry and some incisions

XS: This person made incisions in mice and harvested/processed all tissue for cytokine analysis

MSA: This person helped design experiments and interpret the data

DCY: This person helped design experiments, ran the lab where all cytokine analyses were done and interpreted data.

All listed authors reviewed this manuscript and assisted in its preparation.
